# Effect of preoperative warming combined with dexmedetomidine on postoperative delirium in elderly patients undergoing hip fracture surgery: a randomized controlled trial

**DOI:** 10.3389/fmed.2026.1763529

**Published:** 2026-03-16

**Authors:** Liying Sun, Honglei Wang, Xinnan Ma, Tianwei Tang, Jiayuan Zhai, Songcen Lv, Wanchao Yang

**Affiliations:** 1 Department of Anesthesiology, The Sixth Affiliated Hospital of Harbin Medical University, Harbin, China; 2 Department of Anesthesiology, The Second Affiliated Hospital of Harbin Medical University, Harbin, China; 3 Department of Orthopedics, The Second Affiliated Hospital of Harbin Medical University, Harbin, China

**Keywords:** Dexmedetomidine, hip fracture surgery, Inflammation, postoperative delirium, preoperative warming

## Abstract

**Background:**

We evaluated whether preoperative warming combined with dexmedetomidine reduces postoperative delirium (POD) in older patients undergoing hip fracture surgery.

**Methods:**

This single-blind randomized trial (March–November 2021) enrolled 197 patients aged ≥50 years scheduled for hip fracture surgery. Participants were randomized to warming plus dexmedetomidine (WD), warming alone (W), or control (C). The primary outcome of this manuscript was POD incidence, assessed twice daily from postoperative day (POD) 1 to 3 using the 3D-CAM. Secondary outcomes included delirium days, intraoperative temperature, pain scores (days 1–3), MoCA (days 1 and 3), serum S100β, IL-6, TNF- α, cortisol, and perioperative adverse events.

**Results:**

Of the 174 randomized patients, 153 completed the study and were included in the final analysis. Postoperative delirium occurred in 49.1% of patients in the control group, 26% in the warming group, and 14% in the warming combined with dexmedetomidine group (*P* < 0.001). Delirium duration was significantly shorter in the combined intervention group. Intraoperative body temperatures were consistently higher in the warming and combined groups than in the control group. Postoperative pain scores were significantly lower in the intervention groups. Patients receiving warming combined with dexmedetomidine demonstrated significantly higher postoperative MoCA scores, indicating improved cognitive function. Postoperative inflammatory markers and adverse events were also reduced in the combined group.

**Conclusions:**

Preoperative warming combined with dexmedetomidine was associated with a lower incidence and shorter duration of POD in older patients undergoing hip fracture surgery.

**Clinical Trial Registration:**

Chinese Clinical Trial Registry (ChiCTR2100042142) http://www.chictr.org.cn/showproj.aspx?proj=62146.

## Introduction

Postoperative delirium (POD) is the most common complication of surgery among older patients (1), with an incidence of approximately 50% in those undergoing hip fracture surgeries (2, 3). Patients with hip fractures and delirium have an increased risk of mortality, long-term cognitive impairment, and dementia and a prolonged intensive care unit (ICU) stay (4, 5). Therefore, effective strategies need to be adopted, especially for elderly patients undergoing hip fracture surgery.

However, effective strategies to prevent POD remain lacking. Current prevention methods (6), such as avoiding benzodiazepines, titrating anesthetic depth, and managing pain, are often passive, controversial, and inconsistently effective (7–10). Dexmedetomidine, an α2 adrenergic receptor agonist with sedative and analgesic effects, is increasingly used in older patients (11). Many studies have reported the effect of dexmedetomidine on POD at different time points, such as before anesthesia induction, during surgery, after surgery, and multiple time periods (12, 13). Compared to other sedative agents, dexmedetomidine produces advantageous sedative effects via the endogenous sleep-promoting pathway. These effects include a reduction in opioid dosage, a decreased requirement for anesthesia, and observed neuroprotective effects in animal model studies (14).

The exact pathophysiological mechanism underlying POD remains incompletely understood. Nevertheless, a large-scale observational study has identified a correlation between abnormal intraoperative body temperature and the incidence of POD (15). Additionally, research indicates that up to 44% of elderly patients undergoing hip replacement surgeries may experience perioperative hypothermia (16). Consequently, promoting body temperature is critical for older hospitalized patients, as hypothermia is independently linked to the development of POD (17). Rudiger et al. (18) found that patients having cardiac surgery who developed POD had a lower minimum intraoperative body temperature than those who did not (34.5 °C vs. 35.1 °C). Furthermore, randomized clinical trials have established that preheating patients using forced air heating devices or self-warming blankets effectively mitigates the incidence of hypothermia during the perioperative period (19, 20).

We hypothesized that warming and dexmedetomidine exert a synergistic effect. The combination of these two interventions may reduce postoperative delirium in elderly patients with hip fractures more effectively than either intervention alone. This study aimed to assess the preventive effects of preconditioning alone and the combined approach of preconditioning and dexmedetomidine on the incidence of postoperative delirium in elderly patients with hip fractures.

## Methods

### Design, setting, and ethics

This single-center randomized controlled trial was registered in the Chinese Clinical Trial Registry (ChiCTR2100042142) prior to patient enrolment and was conducted in accordance with the Declaration of Helsinki. Ethical approval was obtained from the institutional ethics committee (Approval No. KY2020-225), and written informed consent was obtained from all participants or their legal representatives.

The registry record describes a two-arm temperature-management trial. However, the study was implemented from initiation as a three-arm randomized controlled trial that included an additional dexmedetomidine group. The randomization sequence, allocation concealment, intervention structure, perioperative management protocols, safety monitoring procedures, and outcome assessment schedule were established prior to enrolment and remained unchanged throughout the trial.

### Participants

The criteria for patient inclusion were defined as follows: (1) aged 50 years or older; (2) classification as American Society of Anesthesiologists (ASA) I or II; and (3) ability to comprehend and provide written informed consent. The exclusion criteria encompassed: (1) presence of one or more comorbidities that could potentially disrupt body temperature regulation; (2) presence of endocrine or metabolic disorders; (3) abnormal coagulation function or recent anticoagulant usage; (4) history of hepatic or renal dysfunction; (5) presence of cardiovascular disease; (6) presence of a significant psychotic disorder impairing cooperation, defined by a mini-mental state examination (MMSE) score < 24; and (7) inability to measure tympanic membrane temperature. Furthermore, patients admitted to the intensive care unit (ICU) postoperatively were excluded from the final analysis. In cases where patients were admitted to the ICU, their clinical management and evaluation were overseen by the ICU physician, leading to the discontinuation of their participation in the trial.

### Randomization and blinding

The participants were randomly assigned in a 1:1:1 ratio to one of three groups:

1. Control group (group C): Patients in the C group did not receive any warming treatment or intravenous dexmedetomidine.
2. Pre-warming group (group W): The patient was warmed using the HICO-AQUATHERM660 physical warming system (Hirtz & Co., KG, Cologne, Germany) at 37 °C for 20 min before anesthesia and maintained at this temperature until the end of operation. Saline was infused at a rate of 0.5 ml kg^−1^ for 10 min before the induction of anesthesia, followed by a continuous infusion at a rate of 0.3 ml kg^−1^ h^−1^ and stopped at the about 30–40 min before the end of the operation.
3. Pre-warming combined with dexmedetomidine group (group WD): The patient was pre-warmed using the HICO-AQUATHERM660 physical warming system (Hirtz & Co., KG, Cologne, Germany) at 37 °C for 20 min before anesthesia and maintained at this temperature until the end of operation. Dexmedetomidine was infused at a rate of 0.5 ml kg^−1^ for 10 min before the induction of anesthesia, followed by a continuous infusion at a rate of 0.3 ml kg^−1^ h^−1^ and stopped at the about 30–40 min before the end of the operation.

This was a single-blind study. Outcome assessors and laboratory personnel were blinded to group allocation, whereas anesthesiologists were not blinded due to the nature of the interventions. Study drugs and saline were prepared by an independent pharmacist and were identical in appearance to maintain blinding of assessors.

### Procedures

In preparation for anesthesia, all patients adhered to a fasting protocol, abstaining from solid foods for 8 h and liquids for 4 h prior to entering the operating theater. No medications related to anesthesia were administered. Upon arrival in the operating room, patients were subjected to standard monitoring procedures, which included electrocardiography, heart rate assessment, non-invasive blood pressure measurement, oxygen saturation monitoring, and body temperature regulation. The ambient temperature within the operating room was maintained at 23 °C.

The induction of anesthesia was achieved using the following pharmacological agents: lidocaine at a dosage of 1–2 mg kg^−1^, sufentanil at 0.3–0.5 μg kg^−1^, propofol at 1–2 mg kg^−1^, and atracurium at 0.3–0.6 mg kg^−1^. During the maintenance phase of general anesthesia, sevoflurane was administered via inhalation at 0.6–0.8 minimum alveolar concentration. Remifentanil was delivered intravenously at a rate of 15–20 μg kg^−1^ h^−1^, along with a concurrent infusion of dexmedetomidine or saline, with adjustments made to maintain a bispectral index between 40 and 60 until the conclusion of the surgical procedure. Atracurium was supplemented at a dose of 0.1–0.2 μg kg^−1^ every 30 min to ensure end-tidal carbon dioxide partial pressure remained within the range of 35–45 mmHg. Intraoperative warming entails positioning a heating blanket on the patient's upper body to provide conductive warming to the arms, shoulders, and back. During the operation, the patient was kept warm until the end of the operation, and their body temperature was continuously and intermittently monitored to ensure a body temperature > 36 °C. Decisions regarding blood transfusion were made collaboratively by the anesthesiologist and the surgeon. Postoperatively, patients were transferred to either the post-anesthesia care unit (PACU) or the intensive care unit (ICU).

### Outcomes

The primary outcome of this analysis (secondary analysis) was the incidence of POD, defined as a state of delirium occurring within the first 3 postoperative days, which was assessed twice daily (8 am and 8 pm) from postoperative day 1 to postoperative day 3 using the 3-min Diagnostic Interview for Confusion Assessment Method (3D-CAM). The 3D-CAM has been confirmed as an efficient method for evaluating delirium in Chinese patients undergoing surgery (21). The registry-listed primary outcomes were thermoregulation-, coagulation-, and recovery-related variables. These outcomes will be reported separately where applicable. In the present manuscript, postoperative delirium (POD) is reported as the primary outcome of a secondary analysis of prospectively collected data from the registered randomized trial. POD was assessed using standardized and predefined assessment procedures during the postoperative period in all randomized participants. All outcome data reported in this manuscript were collected prospectively during trial implementation, and no outcomes were introduced after data analysis had commenced.

### The secondary outcomes were as follows

1. Days with delirium.
2. The body temperature was recorded on arrival in the operating room (T0), before induction of anesthesia (T1), 30min after induction (T2), 1h after induction (T3), 1h after the start of operation (T4), the end of operation (T5), and discharge from the room (T6).
3. Postoperative pain (measured by visual analog scale on postoperative day 1 to 3).
4. Postoperative MoCA scores (Montreal Cognitive Assessment) were recorded (measured by the Montreal scale on 1 and 3 days postoperatively).
5. Perioperative blood levels of inflammatory markers, including S100 calcium-binding protein β (S100β), cortisol, tumor necrosis factor- α (TNF- α) and interleukin-6 (IL-6), were measured preoperatively and on the operative day and postoperative day 1.
6. Record adverse events such as the incidence of hypothermia, Shivering and hypoxemian.

The researchers gathered demographic information from patients, including age, sex, height, weight, years of education, and comorbidities were collected by an investigator during preoperative. Preoperative cognitive function was assessed using the Mini-Mental State Examination (MMSE) (score ranges from 0 to 30).

### Statistical analysis

This study was designed as a pilot, single-blind, randomized controlled trial to explore the feasibility and potential effects of pre-warming alone and pre-warming combined with dexmedetomidine on postoperative delirium in elderly patients undergoing hip fracture surgery. Given the exploratory nature of this pilot study, the sample size was determined based on feasibility considerations during the study period rather than a formal priori power calculation. The primary objective was to obtain preliminary estimates of treatment effects and variability to inform the design of future adequately powered randomized trials.

Statistical analyses were performed using SPSS version 21.0 (SPSS Inc., Chicago, IL, USA). The normality of continuous variables was assessed using the Shapiro-Wilk test. Continuous variables with a normal distribution are presented as mean ± standard deviation (SD), whereas non-normally distributed variables are presented as median with interquartile range (IQR).

For comparisons among the three groups, continuous variables with a normal distribution were analyzed using one-way analysis of variance (ANOVA). When a significant overall difference was detected, *post hoc* pairwise comparisons were performed using the Bonferroni correction. Continuous variables that did not conform to a normal distribution were compared using the Kruskal–Walli's test, followed by pairwise comparisons with the Mann-Whitney U test, with appropriate adjustment for multiple comparisons.

Categorical variables, including the incidence of postoperative delirium and adverse events, are presented as counts and percentages and were compared among groups using the chi-square test or Fisher's exact test, as appropriate. All statistical tests were two-tailed, and a *P* < 0.05 was considered statistically significant.

## Results

### Patients

**Figure 1:**
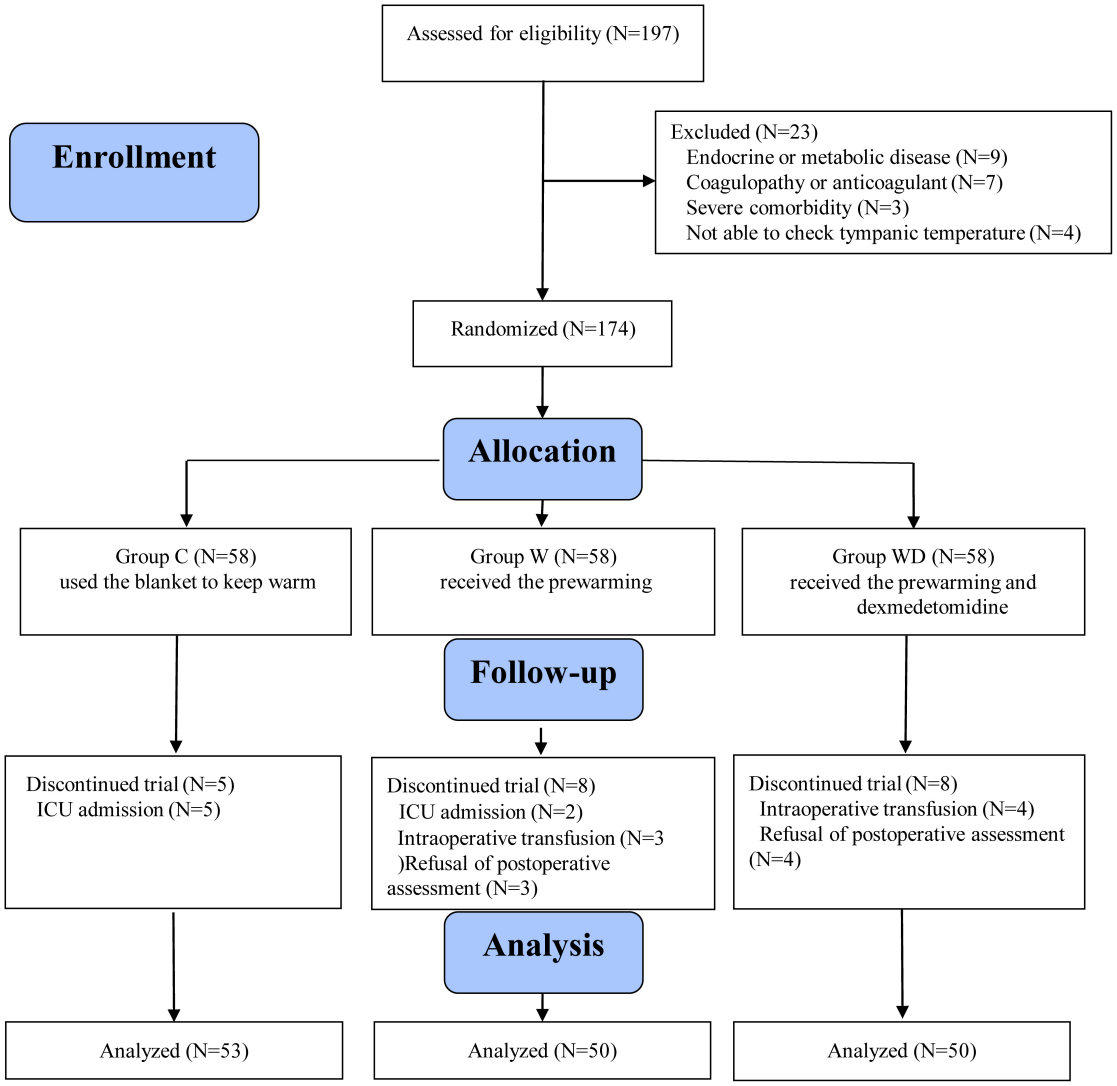
Flow chart of the patients studied.

A total of 197 patients were screened for eligibility between March and November 2021, of whom 174 were randomized. After exclusions due to postoperative ICU admission, intraoperative blood transfusion, or refusal of follow-up assessment, 153 patients were included in the final analysis (53 in group C, 50 in group W, and 50 in group WD), as illustrated in Figure 1.

The baseline characteristics were comparable between the three groups (Table 1).

**Table 1:** Baseline demographic and clinical characteristics.

**Variables**	**C (*n* = 53)**	**W (*n* = 50)**	**WD (*n* = 50)**	***P* value**
**Sex**, ***n*** **(%)**	
Male	24 (45.3%)	15 (30.0%)	24 (48.0%)	0.142
Female	29 (54.7%)	35 (70.0%)	26 (52.0%)	
Age, years	64.2 (8.1)	64.2 (7.2)	64.7 (9.9)	0.928
BMI, kg/m2	24.1 (2.9)	24.5 (4.4)	24.2 (3.0)	0.815
**Years of education**, ***n*** **(%)**	
≤6	27 (50.9%)	20 (40.0%)	24 (48.0%)	0.518
6–9	0 (0%)	0 (0%)	0 (0%)	
≥9	26 (49.1%)	30 (60.0%)	26 (52.0%)	
**Comorbidities**, ***n*** **(%)**	
Hypertension	23 (43.4%)	21 (42.0%)	20 (40.0%)	0.972
Diabetes	8 (15.1%)	6 (12%)	6 (12%)	
Smoking	15 (28.3%)	13 (26.0%)	14 (28%)	
Mini-Mental State Examination	28 (27, 29)	27 (27, 28)	28 (27, 28)	0.177
Preoperative body temperature ( °C), T0	36.8 (0.5)	36.9 (0.5)	36.9 (0.3)	0.655
surgery time (min)	90 (80, 120)	90 (75, 110)	88 (75, 120)	0.739

Continuous variables are expressed as mean ± SD or median [25th, 75th percentiles], as appropriate. Categorical data are presented as the number of events (proportion). BMI body mass index.

### Incidence of POD and days with delirium

Pre-warming alone treatment reduced the incidence of POD from 49.1% in the C group to 26% in the W group. Following the combined administration of dexmedetomidine, the incidence of POD was significantly lower than the W and C groups (*P* < 0.001; Table 2). The WD group was significantly lower than the W and C groups in the number of days positive for delirium (*P* < 0.001; Table 2).

**Table 2:** Primary and secondary outcomes.

**Variables**	**C (*n* = 53)**	**W (*n* = 50)**	**WD (*n* = 50)**	***P* value**
**Primary outcome**	
Postoperative delirium, *n* (%)	26 (49.1%)	13 (26%)^*^	7 (14%)^*#^	<0.001
**Secondary outcomes**	
Days with delirium	0 (0, 1)	0 (0, 1)	0 (0, 0)^*#^	<0.001
**Intraoperative body temperature data**		
T1	36.7 (36.4, 37.0)	36.7 (36.5, 37.0)	36.85 (36.5, 37.1)	0.251
T2	36.4 (36.2, 36.7)	36.7 (36.4, 36.9)^*^	36.5 (36.4, 36.8)^*^	0.01
T3	36.2 (35.9, 36,4)	36.5 (36.3, 36.8)^*^	36.5 (36.3, 36.5)^*^	<0.001
T4	36.1 (35.7, 36.4)	36.5 (36.3, 36.7)^*^	36.4 (36.3, 36.5)^*^	<0.001
T5	36.0 (35.6, 36.2)	36.5 (36.2, 36.6)^*^	36.3 (36.1, 36.5)^*^	<0.001
T6	35.8 (35.3, 36.2)	36.4 (36.0, 36.6)^*^	36.3 (36.2, 36.5)^*^	<0.001
**VAS score**	
Day 1	5 (4, 6)	4 (3, 5)^*^	3 (2, 4)^*^	<0.001
Day 2	3 (2, 4)	2 (2, 3)^*^	2 (1, 3)^*^	<0.001
Day 3	2 (1, 3)	2 (1, 2)	1 (0, 2)^*^	0.002
**MOCA**	
Day 1	25 (24, 26)	26 (24, 27)	27 (26, 28)^*#^	<0.001
Day 3	27 (26, 28)	27 (26, 28)	28 (27, 28.5)^*^	0.006

MoCA, Montreal Cognitive Assessment. ^*^P < 0.05, Significant difference compared with the control group. ^#^*P* < 0.05, Significant difference compared with the W group.

### Intraoperative body temperature, pain scores and MoCA scores

Intraoperative body temperatures at T2, T3, T4, T5, and T6 were significantly higher in the W and WD groups compared with the C group (*P* < 0.001; Table 2). Pain scores were significantly lower in groups W and WD compared to group C on the first and second postoperative days. On the third postoperative day, pain scores in group WD remained lower than those in group C, while no difference was observed between groups W and C (*P* < 0.001; Table 2). MoCA scores in the WD group were significantly higher than those in the W and C groups on postoperative days 1 and 3 (*P* < 0.001, *P* = 0.006; Table 2), indicating better postoperative cognitive performance in patients receiving pre-warming combined with dexmedetomidine.

### Perioperative inflammatory markers

**Figure 2:**
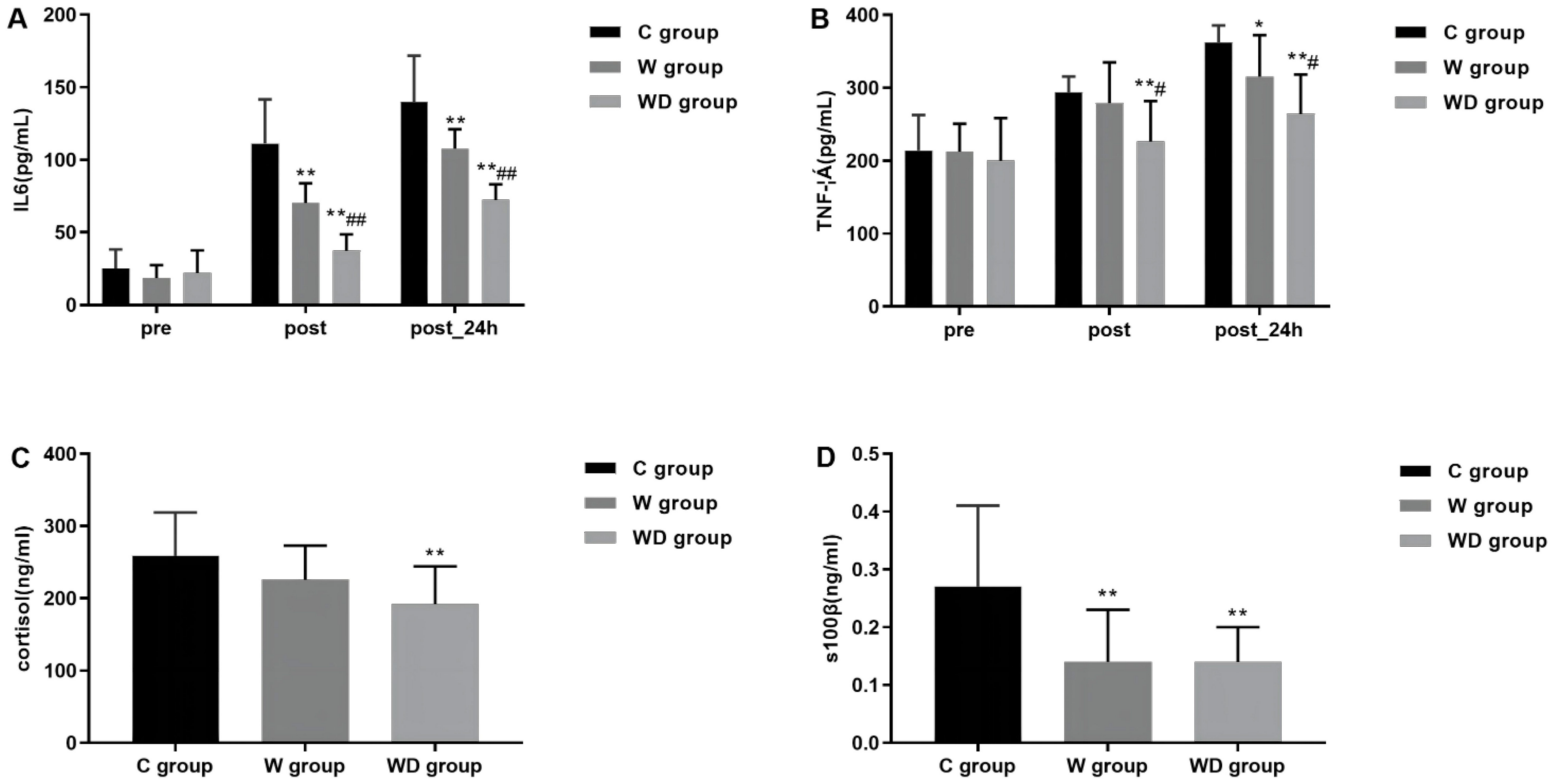
Data are expressed as mean (SD). Compared with group C, the levels of serum IL-6 and TNF- α in group W decreased after operation and 24 h after operation; compared with group W(^*^P < 0.05, ^**^P < 0.01), the levels of serum IL-6 and TNF- α in group WD after operation and 24 h after operation Significantly lower (^#^*P* < 0.05, ^*##*^*P* < 0.01) **(A, B)**. Compared with group C and group W, the levels of plasma cortisol in group WD decreased 24 h after operation(^**^P < 0.01); compared with group C, the levels of serum s100β in group W and group WD decreased 24 h after operation(^**^P < 0.01) **(C, D)**.

At 24 h after operation, serum S100β was statistically and significantly lower in the W and WD groups compared with the C group (*P* < 0.01). Patients in the WD group also had the lowest surgical stress response and inflammatory response, with significantly lower serum IL-6, TNF- α, and plasma cortisol levels at the end of operation and 24 h after operation compared with patients in the C and W groups (*P* < 0.01; Figure 2).

### Adverse events

Pain scores were significantly lower in groups W and WD compared to group C on the first and second postoperative days (*P* < 0.001; Table 3). No cases of hypoxemia were observed during the intervention.

**Table 3:** Adverse events.

**Adverse events**	**C (*n* = 53)**	**W (*n* = 50)**	**WD (*n* = 50)**	***P* value**
Hypothermia, *n* (%)	29 (54.7%)	4 (8.0%)^*^	3 (6.0%)^*^	<0.001
Shivering, *n* (%)	24 (45.3%)	2 (4%)^*^	1 (2%)^*^	<0.001
Hypoxemia, *n* (%)	0 (0. 0)	0 (0.0)	0 (0.0)	

^*^P < 0.05, Significant difference compared with the control group.

## Discussion

This prospective randomized single-blind study aimed to evaluate the preventive effects of preconditioning alone and the combined approach of preconditioning and dexmedetomidine on the incidence of postoperative delirium in elderly patients with hip fractures. The study demonstrated that a perioperative strategy using pre-warming combined with dexmedetomidine decreased the incidence of postoperative delirium (POD) to one-third of that observed in the control group, without any serious adverse events.

POD significantly affects quality of life and is strongly associated with increased morbidity and mortality (4, 5), especially in older patients (22). Elderly patients with hip fracture are at a high risk of POD (23). Our study showed a 49.1 % incidence of POD in the C group, which is consistent with that reported in a previous study (24). The newly reported POD incidence has decreased; however, that observed in our study is within a reasonable range. This difference may be because our patients had a higher incidence of hypothermia and older age in control group. In a large retrospective study of >27,000 patients having noncardiac surgery, Jae-Woo et al. found a significant association between intraoperative hypothermia and the risk of POD. Even patients with mild intraoperative hypothermia (35.0 °C−36.0 °C) had a 1.2–fold higher risk of POD (15). In another study of 22,548 noncardiac surgery patients by Wagner et al. (17) intraoperative hypothermia was independently associated with POD. According to our results, the incidence of POD was significantly lower in group W, which confirms the association between hypothermia and POD. Studies have demonstrated that following the induction of general anesthesia, body temperature typically decreases during the 1 h due to the redistribution of body heat to peripheral tissues (25). The overall intraoperative body temperature was largely determined by the initial decrease in body temperature. In our study, patients in group C exhibited a decline to below 36 °C at 1 h (T3) after anesthesia induction. Michele Carell et al. investigated whether preoperative pre-warming has an effect on intraoperative hypothermia and postoperative functional recovery in ATHA. The findings indicated that preoperative pre-warming effectively delays and reduces heat loss within the 1 h of surgery, enhancing patient comfort and facilitating postoperative functional recovery (26). Consequently, our study implemented pre-warming for patients. The patients in the W and WD groups had stable body temperatures 1h after induction of anesthesia (T3) and intraoperative temperatures were rarely under 36 °C. Thus, pre-warming is crucial.

Dexmedetomidine, an α2 adrenergic receptor agonist with sedative and analgesic effects, is increasingly used in older patients. In a study conducted by J van Norden et al., the impact of dexmedetomidine on POD was examined in both cardiac and noncardiac surgical patients. The findings indicated that among patients aged 60 years and older undergoing major noncardiac and cardiac surgeries, the incidence of postoperative delirium decreased significantly from 44% to 18% (22). Additionally, there was a notable reduction in anxiety on the day of surgery when dexmedetomidine was administered during the perioperative period, compared to placebo. Our study showed that the WD group experienced a 71% relative reduction in the incidence of POD, the shortest duration of delirium, and higher postoperative MoCA scores, which is consistent with these previous observations. [Revised per Reviewer 2] Consistent with our findings, previous clinical and experimental studies have further demonstrated the neuroprotective effects of dexmedetomidine. Chen et al. reported a significant reduction in postoperative delirium following perioperative dexmedetomidine administration in elderly patients, while Zhang et al. demonstrated improved postoperative neurocognitive recovery through attenuation of neuroinflammatory pathways in aged models (27, 28). Prior perioperative studies have also highlighted the role of dexmedetomidine in modulating stress responses and reducing delirium risk in elderly surgical populations. Together, these findings further support the observed benefits of dexmedetomidine when combined with perioperative warming strategies in our study. However, concerns have been raised regarding the potential for dexmedetomidine to affect body temperature regulation, leading to temperature fluctuations (29, 30). We found that the combined use of prewarming and dexmedetomidine had similar effects on perioperative temperature compared to prewarming alone, with dexmedetomidine showing no independent effect on body temperature. It has been suggested that the mechanism by which dexmedetomidine prevents POD may be to exert a sedative effect through endogenous sleep-promoting pathways and to some extent preserve the natural sleep structure and improve sleep quality (31). Studies have shown that cognitive impairment is associated with insomnia and circadian rhythm disturbances (32). A cohort study indicated that while univariate analysis supported the hypothesis that preoperative sleep enhancement decreased the risk of POD, the lack of significance in multivariate analyses suggests that this association may be confounded by other variables, including the effects of the dexmedetomidine intervention itself (33). Consequently, this finding implies that the efficacy of dexmedetomidine in mitigating postoperative delirium may involve mechanisms beyond mere sleep improvement, potentially encompassing its direct neuroprotective or anti-inflammatory properties.

S100β serves as a marker for neurological function associated with brain injury and reflects damage to glial cells (34). Zhao et al. (35) also reported a neuroprotective effect of dexmedetomidine in patients with perioperative hypertensive intracerebral hemorrhage, as evidenced by the inhibition of neuron-specific enolase and S100β levels. Our findings showed that serum S100β levels were significantly decreased in the WD group. This suggests that the combined intervention of prewarming and dexmedetomidine can effectively suppress S100β levels in patients, aligning with previous research outcomes. Several pieces of evidence suggest that systemic or neuroinflammatory responses play an important role in the development of POD (36, 37). Therefore, we chose to administer dexmedetomidine intraoperatively, hypothesizing that it would reduce the incidence of POD by decreasing the inflammatory response. The reduction in the incidence of POD and the significant decrease in postoperative serum IL-6, TNF- α levels and cortisol levels that we observed in the WD group are consistent with the results of previous studies on the effects of dexmedetomidine on the stress response to major surgery, which suggest that dexmedetomidine may improve cognitive function by decreasing the expression and release of inflammatory factors throughout the body, especially in the brain (38, 39).

Previous studies have highlighted the association between postoperative pain, delayed recovery, and POD (40–42). We observed significantly lower pain scores in the W group and WD group compared to the C group. This finding can be attributed to the inhibitory effect of intraoperative pre-warming or dexmedetomidine on the perioperative stress response. Furthermore, the reduction in pain scores may be another reason for the reduced incidence of POD after prewarming or dexmedetomidine treatment.

We acknowledge that our study has several limitations. First, the sample size is small and the single center design. Second, mental status assessment was performed only after a short period of time. Therefore, further prospective studies with larger sample sizes and longer follow-up periods are needed to validate these findings. Finally, we measured inflammatory factors and S100β only 24 h after surgery, as previous research has shown that this time point is critical for capturing the immediate postoperative inflammatory response. Moreover, additional research is warranted to investigate whether prewarming treatment and dexmedetomidine administration impact the functional recovery of the hip following total hip arthroplasty.

In summary, our study shows that general anesthesia with preheating and dexmedetomidine was associated with a reduced incidence of POD in older adult patients undergoing hip replacement surgery. This approach also improves pain control and reduces inflammatory responses post-surgery. These findings inform anesthesia management optimization and patient prognosis enhancement, though further research is warranted.

## Data Availability

The raw data supporting the conclusions of this article will be made available by the authors, without undue reservation.
