# Supplementary material for: Effect of preoperative warming combined with dexmedetomidine on postoperative delirium in elderly patients undergoing hip fracture surgery: a randomized controlled trial
Source: Front Med (Lausanne). 2026 Mar 16;13:1763529. doi: 10.3389/fmed.2026.1763529 (PMC13033707; doi:10.3389/fmed.2026.1763529)
Supplement: Supplementary file 1 [file Table_1.docx]

Supplementary Table S1. Alignment between registry record (ChiCTR2100042142) and implemented trial protocol

| **Item** | **Registry entry (ChiCTR)** | **Implemented in this trial/manuscript** | **Timing / documentation** | **Rationale** | **Potential impact & mitigation** | **Where disclosed** |
| --- | --- | --- | --- | --- | --- | --- |
| Registration number | ChiCTR2100042142 | ChiCTR2100042142 (same record cited) | Prospective registration (per registry); manuscript submitted thereafter | Provide traceability to registry record | — | Abstract; Methods; Supplementary Table S1 |
| Study timeframe | Study execute time: 2020-12-01 to 2021-12-01; Recruiting time: 2021-01-15 to 2021-12-31 | Trial conducted March–November 2021 in the manuscript; recruitment and follow-up shown in CONSORT flow. | Registry timestamps vs manuscript study period | Registry fields capture planned execute/recruit periods; manuscript reports the actual study conduct window. | Low risk; clarified explicitly; CONSORT flow provided | Methods; flow chart |
| Population / procedure wording | Joint surgery context (per registry title/fields in screenshots) | Elderly patients undergoing hip fracture surgery (including hip arthroplasty or internal fixation as clinically indicated) | Clarified wording in Methods; surgery type per clinical indication | Align disease context (hip fracture) while covering procedure variations | Improves consistency; reduces ambiguity | Title/Abstract/Methods; Keywords |
| Design / number of arms | Two-arm design displayed: 1) Intraoperative insulation group — sample size 35 2) Preinsulation + intraoperative insulation— sample size 35 | Three-arm randomised trial from initiation: Control (C); Warming/insulation (W); Warming/insulation + dexmedetomidine (WD). | Implemented as 3-arm at trial initiation; registry display not updated to reflect this refinement. No separate ethics amendment submitted. | Dexmedetomidine arm included at trial initiation (before first participant enrolled) | Not interim-result driven; randomisation/allocation concealment unchanged; uniform safety monitoring and outcome assessment across groups. | Abstract (Trial registration); Methods (Design/Ethics); Response to Reviewers; Supplementary Table S1 |
| Interventions | Arm 1: Intraoperative insulation. Arm 2: Pre-insulation + intraoperative insulation. | Warming/insulation procedures as described, plus dexmedetomidine regimen in WD arm (standard clinical dosing). Perioperative care otherwise standardised. | Protocol/SOP; anaesthesia records; drug preparation log | Evaluate combined strategy (warming + dexmedetomidine) | Safety monitoring applied uniformly; adverse events recorded and reported. | Methods (Interventions); Results |
| Registry-listed primary outcomes / objective | Primary outcomes (Type: Primary) shown in screenshots: • Degree of shivering • Incidence of surgical site infection • Number of days in hospital • Coagulation function (Measure method: Thrombus elastograph / TEG) • Inflammatory factors • Wake up time Measure time point: postoperative assessment only; exact time points not specified in the registry record. | Postoperative delirium (POD) reported as main outcome of a secondary analysis of prospectively collected data, assessed using 3D-CAM twice daily on postoperative days 1–3. Other reported outcomes: delirium days, intraoperative temperature, pain (VAS POD1–3), MoCA (POD1 & POD3), S100β, IL-6, TNF-α, cortisol, and perioperative adverse events. | POD assessment plan finalized before enrollment | Clinically relevant neurocognitive endpoint in older hip fracture patients; POD data were collected prospectively within the same study framework. | Explicit transparency statements added; standardized (assessor-blinded) POD assessment schedule; registry–manuscript mapping provided (Supplementary Table S1) to minimise concerns about selective reporting. Because the registry did not specify exact time points, we transparently report the actual assessment schedule used in this manuscript. | Abstract (Trial registration); Methods (Outcomes); Discussion (Transparency statement); Supplementary Supplementary Table S1 |
| Sample size / analysis population | 35 per arm displayed (2 arms) | 197 screened; 174 randomised; 153 analysed (mITT/available POD assessments) across 3 arms | CONSORT flow; prespecified POD assessment schedule (POD1–POD3) | Feasibility and assessability of POD outcomes | Disclose reasons for non-assessability (e.g., ICU transfer); report flow diagram; consistent assessment schedule. | Results (Flow diagram); Methods (Analysis set); Supplementary Table S1 |
| Eligibility criteria | Screenshots show eligibility fields (not fully visible in provided images) | Inclusion: age ≥50, ASA I–II, informed consent; exclusion: comorbidities affecting thermoregulation, endocrine/metabolic disorders, abnormal coagulation/anticoagulants, hepatic/renal dysfunction, cardiovascular disease, severe psychiatric disorder (MMSE<24), inability to measure tympanic temperature. | Screening log; case report forms | Standardise baseline risk and feasibility of temperature monitoring | Report baseline comparability across groups; CONSORT flow | Methods (Participants); Table 1; flow chart |
| Randomisation / blinding | Not fully visible in provided registry screenshots | Computer-generated randomisation; allocation concealment per manuscript; assessor-blinded (single-blind) outcome assessment (3D-CAM). | Randomisation list; sealed envelopes/central system (as applicable). Same randomization sequence and allocation concealment applied to all three groups | Preserve internal validity | Blinded assessors reduce detection bias; standardised procedures across arms. | Methods (Randomisation/Blinding) |
| Ethics / consent | Ethics-related fields present in registry (details not fully visible in screenshots) | Ethics approval: KY2020-225; written informed consent obtained. No separate ethics amendment submitted for the design refinement; trial conducted under institutional ethics oversight with uniform safety monitoring. | Ethics approval letter; consent forms; AE monitoring plan | Ensure participant protection and oversight | Safety monitoring and AE reporting applied uniformly; transparency in reporting deviations. | Methods (Ethics); Response to Reviewers; Supplementary Table S1 |

Abbreviations: POD, postoperative delirium; 3D-CAM, 3-Minute Diagnostic Interview for CAM; TEG, thromboelastography; mITT, modified intention-to-treat; AE, adverse event.

Additional note: All outcomes reported in the present manuscript were prospectively collected during trial implementation under the same ethics approval, recruitment period, and follow-up schedule as described in the registry.
